# “A Picture Paints a Thousand Words”—A Systematic Review of the Ethical Issues of Prenatal Ultrasound

**DOI:** 10.1007/s11673-024-10360-0

**Published:** 2024-08-19

**Authors:** M. Favaretto, M. Rost

**Affiliations:** Institute for Biomedical Ethics, University of Basel, Bernoullistrasse 28, 4056 Basel, Switzerland

**Keywords:** Ultrasound, Ethics, Review, Obstetrics, Fetus, Pregnancy, Midwifery

## Abstract

Prenatal ultrasound is a non-invasive diagnostic examination. Despite the recognized diagnostic value, this technology raises complex ethical questions. The aim of this study is to provide a comprehensive analysis that coherently maps the ethical challenges raised by prenatal ultrasound examination, both 2D and 3D. We performed a systematic literature review. Six databases were systematically searched. The results highlight how concerns related to beneficence, informed consent, and autonomy are mainly related to routine use of prenatal ultrasound in the clinical context, while considerations linked to overmedicalization of pregnancy, discrimination, and fetal ontology were often reported in relation to the impact ultrasound is having on medicine and society. Ethical issues in the context of pre-abortion ultrasound, obstetric practices in low-income settings, and keepsake ultrasound imaging were also greatly discussed. Since prenatal ultrasound practices critically impact pregnant people’s autonomy and their role within pregnancy, we conclude that information providing strategies should be developed to appropriately inform pregnant people about the nature, purpose, risks, and choices revolving around prenatal ultrasound. In addition, as it is becoming increasingly difficult to separate the social and clinical dimensions of prenatal ultrasound, future research should focus on examining if and how such dimensions should be reconciled.

## Introduction

Obstetric ultrasonography, or prenatal ultrasound, is a commonly used technology in obstetrics. It is a medical examination under the category of diagnostic imaging. Traditional B-Mode (or 2D) ultrasound utilizes basic acoustic principles to produce luminosity patterns that allow the provider to visualize basic structures and organs within the belly (fetal structure, fetal organs, fetal heartbeat, uterine artery, etc.) and assess their conformity to the regular presentation of the examined structure (Semczyszyn 2010). Three dimensional ultrasound, a technological development of traditional ultrasound, offers a clearer three-dimensional picture of the fetus and allows for more sophisticated visualization in ways B-Mode imaging technology could not accomplish. Particularly, surface rendered images (showing the surface of the fetus) are significantly easier to read and interpret for providers and expectant parents (Roberts 2012).

The diagnostic value of prenatal sonograms is well recognized (Levy and Stosic 2019) and ultrasound has been increasingly utilized in obstetric care for several decades, so much that it is now considered a routine pregnancy examination in a growing number of countries (Åhman et al. 2019). The main purpose of ultrasound is to monitor the fetal health, estimate gestational age and amniotic fluid amount, and detect multiple pregnancies (Whitworth et al. 2015). Thanks to the technological sophistication and development of prenatal ultrasound, it is now possible to diagnose a wide range of medical conditions, from fetal malformations to genetic conditions (Curado and Bhide 2018). Three dimensional ultrasound, on the other hand, is being currently assessed in its usefulness in clinical practice, particularly for diagnostic evaluation of fetal malformations (Sepulveda et al. 2012). Although 3D ultrasound leads to better visualization of fetal anatomy and improves the detection rate of structural fetal abnormalities, it only complements, rather than substitutes for, conventional B-Mode (Kurjak, Azumendi et al. 2007a). In addition, the provider dependence associated with this technology can lead to inaccuracy in evaluation if not used by a highly trained provider (Kurjak, Miskovic et al. 2007a; Markov et al. 2010). Despite this, knowledge on fetal development and behaviour has been promoted by the concurrent development of prenatal ultrasound (Ramón y Cajal and Martínez 2005; Alty and Hoey 2013).

Although classified as a non-invasive procedure and seen as involving minimal risk (Abramowicz 2013) as a medical diagnostic technology, ethical appraisal of ultrasound has been performed within common ethical frameworks in bioethics (Beauchamp and Childress 2001). In this context, ethical considerations surrounding prenatal ultrasound seem to be mainly related to properly balancing the benefits and risks of the technology together with safeguarding pregnant people’s autonomy (Chervenak and McCullough 1991). In addition, because of its increased use throughout the years, experts have been interrogating themselves about the impact of establishing prenatal ultrasound as a routine examination (Lowe et al. 1998; Edvardsson et al. 2018). At the same time, however, ultrasound is different from most reproductive technologies and other diagnostic imaging examinations. Prenatal ultrasound is considered a technology of reassurance for pregnant people regarding the appropriate course of pregnancy (Thomas et al. 2017), and typically, ultrasound images have a life outside of the clinic, as they are generally well appreciated by expectant parents who want to view the fetus during pregnancy. Fetal scans are shared with family and friends and displayed in homes and offices (Roberts 2012). Even more, sophistications in ultrasound technology like 3D allow for enhanced visualization of the fetus inside the womb, including the possibility of seeing the facial features (Mills 2011). With 2D ultrasound, images are often not recognizable by lay people, and a provider is needed to guide the parents in recognizing the features of their child. With 3D imaging, parents are able to recognize the fetus independently, including identifying its face and other features (Roberts et al. 2015).

As a sophisticated visual medium, ultrasound technology therefore raises additional ethical issues. For instance, it has been argued that the increased use of ultrasound from the early stages of pregnancy onwards has promoted the conceptualization of the fetus as a person (Giebel 2020). As a consequence, personification of the fetus has led to the identification of the fetus as an individual patient (Zechmeister 2001; Edvardsson, Small et al. 2015b). In addition, there are many concerns regarding the impact that visualization of the fetus may have on decision-making processes in cases where fetal and the pregnant person’s interests are not aligned (Bijma et al. 2008). Finally, it has also been highlighted how enhanced 3D imaging might have such a strong impact as to challenge society’s position on critical matters such as abortion, care, patients’ autonomy, and personal identity (Gilbert and Howes-Mischel 2004; Mills 2011). Although some of these issues have been touched on by the literature in bioethics and feminist studies, a comprehensive and systematic evaluation of such ethical considerations is missing. Moreover, scarce investigation has been carried out regarding the role and specific issues of 3D ultrasound. Thus, this systematic review comprehensively and coherently maps the ethical issues and considerations associated with prenatal ultrasound and its routine use in clinical practice.

## Methods

### Search Methodology

**Fig. 1:**
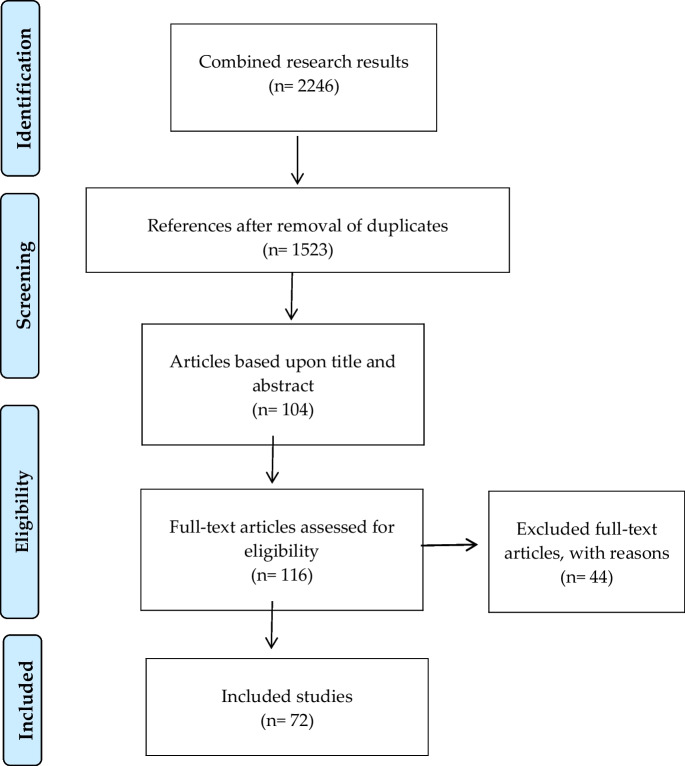
PRISMA flowchart

We performed a systematic literature review by searching six databases, using Boolean logic (Table 1). Preferred Reporting Items for Systematic Reviews are presented in Fig. 1 (Moher et al. 2015).

**Table 1:** Search terms and results

	Search terms	Pub Med	Web of Science	Soc Index	CINAHL	Phil papers	Psych Info
1	(prenatal OR obstetric* OR fetal)	782,856	652,865	10,454	132,389	1,496	27,592
2	(ultrasound OR sonograph* OR ultrasonograph* OR scan*)	1,983,109	2,210,882	9,687	223,748	3,486	37,741
3	(ethic* OR moral*)	299,411	391,612	111,601	107,576	224,709	75,990
	1 AND 2 AND 3	**1,219**	**659**	**19**	**290**	**31**	**28**

Date of last search: May 14, 2021

### Inclusion and Exclusion Criteria

We included papers in English with no restriction on the type of methodology (quantitative, qualitative, mixed methods, theoretical) or time of publication. We only included original research articles and opinion papers from peer-reviewed journals. Grey literature, books, conference proceedings, dissertations, posters, letters, editorials, and introductions to special issues were omitted.

### Search Results and Data Extraction

After deduplication (n=723), 1523 papers remained. In the next phase, we included all articles that enumerated, described, or discussed the ethical issues of prenatal ultrasound—both 2D and 3D. We only included papers that explicitly discussed ethical dilemmas or that flagged social and political dimensions, personal views, or any other aspect explicitly as an ethical dimension. Consequently, papers that focused on communication between patients and providers or on the personal opinions of pregnant people or providers about ultrasound were not included. In addition, papers that focused exclusively on the ethical issues of medical conditions detected via ultrasound (e.g., Down syndrome) were excluded. Additional papers (thirteen) were excluded because they were editorials, letters, case studies, commentaries, or not in English. In total 1419 papers were excluded in this step.

Subsequently, we scanned the references of the remaining 104 articles to identify additional studies, resulting in twelve added papers. This resulted in 116 articles selected for full-text review. During the next phase, MF read the full texts. After thorough evaluation, forty-four articles were excluded for the following reasons: they 1) were retracted after publication; 2) did not explicitly address ethical dimensions specific to ultrasound; 3) were a book chapter, literature review, case study, editorial, or letter that was not previously identified; 4) did not focus on ultrasound but on other reproductive technologies; 5) were written in a language other than English. Uncertainties were discussed with MR who evaluated the articles’ eligibility based on their abstracts and full texts. Once consensus was reached, the articles were included. Finally, we analysed the remaining seventy-two articles. We extracted the following information: year and country of publication, methodology, specific field of application of ultrasound, ethical issues that emerge from ultrasound, mention of 3D ultrasound and of specific ethical issues.

## Results

Most (forty-seven) papers were theoretical. The remaining twenty-five papers employed empirical methods (twenty-four qualitative, one quantitative). Most papers (twenty-seven) were from the United States, eleven from the United Kingdom, five from Australia, and three from the Netherlands. Eleven papers were from multiple countries. The majority of the papers discussed prenatal ultrasound screening in general, while other papers discussed specific applications of ultrasound, such as screening for fetal abnormalities, genetic disorders, soft markers, fetal diseases, or Down syndrome; pre-abortion ultrasound; prenatal screening for sex identification; and keepsake ultrasound. A few papers talked about ultrasound within a discussion on ultrasound images (Table 2). Only sixteen mentioned 3D and 4D ultrasound. Among these, only eight papers discussed ethical issues related to this technology (Zechmeister 2001; Sullivan 2002; Burlbaw 2004; Voelker 2005; Mills 2008; Dowdy 2016; Mills 2018; Favaretto et al. 2020).

**Table 2:** List of included papers

Author and year	Country	Design	Participants^1^	Specific application
Åhman et al. (2019)	Sweden/ Australia	Empirical, qualitative	A sample of midwives in Norway	Routine prenatal ultrasound
Ahman et al. (2010)	Sweden	Empirical, qualitative	A sample of pregnant women	Screening for identification of soft markers
Åhman et al. (2015)	Norway/Sweden	Empirical, qualitative	A sample of obstetricians	Routine ultrasound
Aune and Möller (2012)	Norway/Sweden	Empirical qualitative	A sample of pregnant women	Screening for risk assessment of chromosomal abnormalities
Bashour et al. (2005)	Syria/Lebanon	Empirical, qualitative	A sample of pregnant women	Routine prenatal ultrasound
Bijma et al. (2008)	Netherlands	Theoretical		Screening for structural abnormalities
Boucher (2004)	USA	Theoretical		Ultrasound images
Brauer (2016)	Switzerland	Theoretical		Routine prenatal ultrasound
Burlbaw (2004)	USA	Theoretical, opinion		Keepsake ultrasound imaging
Burrow (2012)	Australia	Theoretical		Routine prenatal ultrasound
Charo (2014)	USA	Theoretical, opinion		Pre-abortion ultrasound
Chasen and Skupski (2003)	USA	Theoretical		Screening for Down syndrome
Chervenak and McCullough (1996)	USA	Theoretical, opinion		Routine prenatal screening for sex identification
Chervenak and McCullough (2005)	USA	Theoretical		Keepsake ultrasound imaging
Chervenak and McCullough (1989)	USA	Theoretical		Routine prenatal ultrasound
Chervenak et al. (1989)	USA	Theoretical		Routine prenatal ultrasound
Coles (2007)	UK	Theoretical, opinion		Keepsake ultrasound imaging
Curado and Bhide (2018)	Netherlands	Theoretical		Screening for structural abnormalities
de Jong et al. (2011)	Netherlands	Theoretical		Screening for fetal abnormalities
Donovan (2006)	New Zealand	Empirical, qualitative	A sample of pregnant women	Routine prenatal ultrasound
Dowdy (2016)	USA	Theoretical		Keepsake ultrasound imaging
Edvardsson et al. (2018)	Sweden/Australia/Norway	Empirical, qualitative	A sample of Norse obstetricians	Routine prenatal ultrasound
Edvardsson et al. (2016)	Sweden/Australia	Empirical, qualitative	A sample of Swedish midwives	Routine prenatal ultrasound
Edvardsson, Small et al. (2015b)	Sweden/Australia	Empirical, qualitative	A sample of Australian obstetrics	Routine prenatal ultrasound
Edvardsson et al. (2014)	Sweden/Australia	Empirical, qualitative	A sample of Australian obstetrics	Routine prenatal ultrasound
Edvardsson, Mogren et al. (2015a)	Sweden/Australia	Empirical, qualitative	A sample of Australian midwives	Routine prenatal ultrasound
Edwards and Thomson (2012)	UK	Theoretical		Ultrasound for sex determination
Favre et al. (2009)	France	Empirical, quantitative	A sample of medical correspondents	Screening for Down syndrome
Favaretto et al. (2020)	Belgium	Theoretical		Ultrasound images
Gammeltoft and Nguyen (2007)	Denmark/Vietnam	Empirical, qualitative	A sample of physicians	Routine prenatal ultrasound
Getz and Kirkengen (2003)	Norway/Iceland	Theoretical		Screening for risk assessment of chromosomal abnormalities
Giebel (2020)	USA	Theoretical		Ultrasound images and abortion
Gorincour et al. (2011)	France	Theoretical		Ultrasound images and abortion
Gottfreosdottir and Bjornsdottir (2010)	Iceland	Empirical, qualitative	Newspaper articles and other public media	Screening for Down syndrome
Graham et al. (2010)	UK	Empirical, qualitative	A sample of women seeking abortion; a sample of sonographers	Pre-abortion ultrasound
Hemminki et al. (2000)	Finland	Empirical, qualitative	A sample of physicians	Screening for fetal abnormalities
Holmlund et al. (2020)	Sweden/Vietnam/ Australia/Rwanda	Empirical, qualitative	A sample of Vietnamese midwives	Routine prenatal ultrasound
Howe (2014)	UK	Theoretical		Routine prenatal ultrasound
Kongnyuy and van den Broek (2007)	UK	Theoretical		Routine prenatal ultrasound
Leung et al. (2015)	China	Theoretical		Keepsake ultrasound imaging
Lowe et al. (1998)	USA	Theoretical		Routine prenatal ultrasound
McFadyen et al. (1998)	UK	Theoretical, opinion		Routine prenatal ultrasound
Mills (2005)	Australia	Theoretical		Ultrasound images
Mills (2008)	Australia	Theoretical		Ultrasound images
Mills (2018)	Australia	Theoretical		Pre-abortion ultrasound
Minkoff and Ecker (2012)	USA	Theoretical, opinion		Pre-abortion ultrasound
Mubuuke (2011)	Uganda	Empirical, qualitative	A sample of sonographers; a sample of pregnant women	Ultrasound for sex determination
Paul and Nawrocki (1997)	USA	Theoretical		Keepsake ultrasound imaging
Petchesky (1987)	USA	Theoretical		Ultrasound images
Phutke et al. (2019)	India/USA	Theoretical		Routine prenatal ultrasound
Raucher (2009)	USA	Theoretical		Keepsake ultrasound imaging
Rocha (2012)	USA	Theoretical		Pre-abortion ultrasound
Sandelowski (1994)	USA	Empirical, qualitative	A sample of pregnant couples	Routine prenatal ultrasound
Sandelowski (1998)	USA	Theoretical		Routine prenatal ultrasound
Sanger (2008)	USA	Theoretical		Routine prenatal ultrasound/Pre-abortion ultrasound
Simonsen et al. (2008)	USA	Theoretical, opinion		Keepsake ultrasound
Skupski et al. (1994)	USA	Theoretical		Routine prenatal ultrasound
Steinauer and Sufrin (2014)	USA	Theoretical		Pre-abortion ultrasound
Stephenson et al. (2017)	Australia	Empirical, qualitative	A sample of obstetric specialists	Routine prenatal ultrasound
Stormer (2003)	USA	Theoretical, opinion		Routine prenatal ultrasound
Sullivan (2002)	USA	Theoretical		Routine prenatal ultrasound
Taylor (2000)	USA	Theoretical		Routine prenatal ultrasound
Taylor (2002)	USA	Theoretical		Ultrasound images
Thomas et al. (2017)	UK	Empirical, qualitative	A sample of pregnant women; a sample of practitioners	Routine prenatal ultrasound
Van der Zalm and Byrne (2006)	Canada	Empirical, qualitative	A sample of pregnant women	Routine prenatal ultrasound
Verbeek (2008)	Netherlands	Theoretical		Routine prenatal screening
Voelker (2005)	USA	Theoretical, opinion		Keepsake ultrasound
Williams et al. (2002)	UK	Empirical, qualitative	A sample of practitioners in the area of perinatal care	Screening for Down’s syndrome
Williams (2006)	UK	Empirical, qualitative	A sample of practitioners	Routine prenatal ultrasound
Whitbeck (1987)	UK	Theoretical		Routine prenatal ultrasound
Yuen et al. (2006)	China	Theoretical		Screening for fetal diseases
Zechmeister (2001)	Austria	Theoretical		Routine prenatal ultrasound

^1^Original terminology of respective authors

### Ultrasound Screening and Ethical Issues in the Clinical Context

#### Beneficence, Harm, and Risk Assessment

Among the papers that discussed routine prenatal ultrasound, seventeen mentioned possible harm that could derive from its use for both expectant parents and the fetus. In this context, anxiety or adverse psychological effects were flagged as possible harmful consequences for pregnant people (and their partners) that could be caused by routine performance of ultrasound examination and its potential outcomes (Whitbeck 1987; Lowe et al. 1998; Van der Zalm and Byrne 2006; Åhman et al. 2015; Curado and Bhide 2018), even in studies where the technology was described as a tool for reassurance (Thomas et al. 2017).

Numerous papers, especially the ones focusing on screening for fetal diseases, related increased parental anxiety and profound distress for pregnant people, such as negative feelings associated with psychological trauma, in response to adverse ultrasound findings (Edvardsson, Mogren et al. 2015a), detection of soft markers (Getz and Kirkengen 2003; Ahman et al. 2010), fetal anomalies (Bijma et al. 2008; Gottfreosdottir and Bjornsdottir 2010), or generally anxiety-producing information based on ultrasound scans (Bashour et al. 2005). According to Aune and Möller (2012), anxiety and feeling of guilt are particularly strong in case of decision-making scenarios. Parental distress was also considered to be a result of uncertain (Sullivan 2002; Edvardsson et al. 2016) or false (Chervenak et al. 1989) ultrasound findings and the inadequate competence by providers performing the ultrasound (Skupski et al. 1994) that could lead to inadequate readings or poor communication (Howe 2014).

In addition, a couple of papers linked parental emotional stress to the visualization of the fetus through the ultrasound screen. According to Åhman et al. (2019), for instance, the intense use of ultrasound and the visualization of the fetus through the screen promotes the view of the fetus as vulnerable, thus possibly increasing anxiety and guilt in pregnant people. Edvardsson et al. (2018) associated visualization of the fetus with increased emotional stress in cases where the pregnant person had seen numerous images of an apparently healthy fetus but subsequently experienced an abortion.

Harm to the fetus was discussed mostly in relation to the difficulty to balance the risks and benefits of ultrasound. A couple of papers mentioned the lack of evidence that completely excludes harm to the fetus deriving from exposure to the high-frequency sound waves involved in the procedure (Chervenak et al. 1989; Taylor 2002; Holmlund et al. 2020). Two other papers highlighted how some uncertain findings of the ultrasound could only be confirmed by additional invasive tests and procedures, such as amniocenteses, that could harm the fetus (Chasen and Skupski 2003; Williams 2006; Verbeek 2008).

#### Information Provision and Informed Consent Practices

Numerous papers focused on the ethical dilemmas deriving from information provision, prenatal counselling, and informed consent practices related to prenatal ultrasound. Some papers described ultrasound as a routine diagnostic examination that might undermine and neglect adequate practices of informed consent, as it has been developing throughout the years as a procedure where counselling is either dispensable or not needed (McFadyen et al. 1998; Brauer 2016). Moreover, according to some of the retrieved papers, pregnant people are often not appropriately informed by providers about the nature of the screening, the purpose, and the risks and benefits entailed by ultrasound (Lowe et al. 1998; Favre et al. 2009; Thomas et al. 2017), despite the fact that ultrasound is a technology that could reveal severe fetal abnormalities or other critical fetal issues (Sanger 2008).

The absence of appropriate procedures for informed consent was considered problematic, as pregnant people may lack the knowledge required to make informed decisions regarding prenatal diagnosis (Edvardsson et al. 2016). In addition, poor counselling practices were argued to hinder decision-making for pregnant people, as they would limit the possibility for pregnant people to make informed choices about further diagnostic tests (de Jong et al. 2011; Edvardsson, Mogren et al. 2015a). According to the retrieved literature, informed consent might be undermined by various factors. First, consent might be hindered in case of incomplete or inaccurate reporting of results to the pregnant person (Skupski et al. 1994). Second, according to a study by Edvardsson, Mogren et al. (2015a), providers might find it challenging to communicate complex information to expectant parents with low levels of education, who are non-English speaking or from other cultural settings, or that suffer from mental disabilities. Third, providers might have difficulties in transforming anatomical images into clear, meaningful information about a fetus and appropriately giving the patient adequate counselling (Stephenson et al. 2017). It was also argued that pregnant people have difficulties in understanding the true meaning of risk assessment, as ultrasound is viewed by parents as a time to see the baby or to have confirmation of pregnancy (Williams et al. 2002; Aune and Möller 2012). Finally, Sandelowski (1998) argued that informed consent might be undermined when providers allow couples to understand fetal ultrasonography solely as a bonding moment with their babies.

In this context, three papers stressed the importance of promoting adequate informed consent processes in obstetric ultrasound (Chervenak and McCullough 1989), such as making the parents aware of the complexity of ultrasound (Gorincour et al. 2011), together with determining the underlying causes for pregnant people being inadequately informed (Edvardsson, Mogren et al. 2015a). Finally, two papers focused on the difficulties and dilemmas of providers in appropriately deciding what information to provide to expectant parents. According to Edvardsson et al. (2014), disclosing all the information to expectant parents might be counterproductive as it might increase unwarranted worry or anxiety. In addition, uncertainty of findings and ambiguities in ultrasound outcomes create difficulties for providers in drawing a clear line for what information should be provided (Edvardsson et al. 2016).

#### Autonomy, Agency, and Decision-Making

A considerable number of papers associated issues of autonomy with ultrasound. Among them, some papers argued that routinizing and normalizing ultrasound could hinder pregnant people’s choice, as the examination is generally perceived as an integral part of prenatal care that is necessary for a good pregnancy outcome (Aune and Möller 2012). Parents, in this scenario, might receive subtle or manifest societal pressure to conform to community expectations of undergoing ultrasound and other tests in pregnancy (Edvardsson, Mogren et al. 2015a; Edvardsson et al. 2018), thus undermining pregnant people’s ability to independently choose the use of the technology and erasing the possibility of an unexamined pregnancy as a sanctioned option (Donovan 2006; Burrow 2012; Stephenson et al. 2017).

Other papers argued that while ultrasound screening is considered to promote individual freedom of choice, the technology tends to circumscribe pregnant people’s decision-making about their pregnancies to a fixed number of tests, therefore making it difficult to safeguard autonomy (Favre et al. 2009; Åhman et al. 2015). A couple of papers focused on the effect of ultrasound of dispossessing pregnant people from their subjective experience over pregnancy and their agency and opportunity to act independently on behalf of the fetus (Whitbeck 1987; Brauer 2016). Some of the identified articles highlighted how ultrasound technology might also challenge and complicate parental decision-making. As four papers pointed out, ultrasound has the potential to generate huge amounts of information that might cause difficult decision-making in maternity care or put pregnant people in very difficult, or even unbearable, situations (Edvardsson et al. 2016; Edvardsson et al. 2018; Phutke et al. 2019), such as deciding whether to electively terminate a desired pregnancy when particular genetic conditions or malformations are discovered (Taylor 2002). Some papers highlighted that decision-making is made even more arduous in case of uncertainty regarding the significance of the findings, when the expectant parents are faced with probability estimates regarding fetal outcomes (Williams 2006; Edvardsson et al. 2014; Edvardsson, Mogren et al. 2015a; Edvardsson, Mogren et al. 2015a), and when it is not clear what kind of tests to take forward, or if abortion is recommended (Stephenson et al. 2017; Thomas et al. 2017).

Four papers related complications of parental decision-making to the visualization of the fetus through the ultrasound scan, as it might have an impact on decisions regarding continuation of pregnancy, abortion, and possibly harmful follow-up tests (Chervenak and McCullough 1989; Verbeek 2008; Aune and Möller 2012; Favaretto et al. 2020). In this context, some of the retrieved literature also identified ethical issues within obstetric ultrasound in the creation of diverging interests between the pregnant person and the fetus. According to a few papers, ultrasound technology has the capacity to put the focus on the fetus and its health (Åhman et al. 2015; Åhman et al. 2019) by visually separating it from the pregnant person (Brauer 2016), thus influencing the balancing of a pregnant person’s and fetal well-being and bringing out conflicts between the two sets of interests (Burrow 2012; Edvardsson, Mogren et al. 2015a).

### Ultrasound’s Impact on Pregnant People, Society and Fetal Ontology

#### Overmedicalization of Pregnancy, Spectacularization, and Disembodiment

The retrieved literature also discussed the impact that the routine and widespread use of ultrasound has on pregnancy and the obstetric practice. Some papers argued how ultrasound has contributed to increased medicalization of pregnancy (Whitbeck 1987; Edvardsson, Mogren et al. 2015a). In this context, ultrasound becomes a form of medical surveillance that closely monitors pregnancy from the early stages (Brauer 2016) or a maximization of medical control over pregnancy (Petchesky 1987) in a society where natural birth is not an option for families as they must undergo a large number of antenatal examinations (Favre et al. 2009).

Other papers focused on the role that ultrasound technology has in spectacularizing and commodifying pregnancy and obstetric care. According to two papers by Sandelowski (Sandelowski 1994; Sandelowski 1998), imaging technologies have a tendency to spectacularize care and nursing, and specifically ultrasound has the capacity to create a mediated reality that makes pregnancy a public spectacle. Sullivan (2002) similarly argued that ultrasound technology has become a spectacle rather than a diagnostic tool. Additional papers focused instead on the aesthetic consumerism that ultrasound technology has brought upon obstetric care (Whitbeck 1987; Sanger 2008). In this context, the papers argued that ultrasound has become a consumeristic commodity where pregnant people consume fetal images (Zechmeister 2001) and the fetus is “produced” as an object for exchange and consumption (Taylor 2000). Sullivan (2002) also argued that the advent of 3D ultrasound has exacerbated the trivialization of ultrasound technology.

Some papers also raised ethical issues in relation to the impact that the advancement of ultrasound technologies has on pregnant people. Sandelowski (Sandelowski 1994; Sandelowski 1998) described how ultrasound forces pregnant people to experience pregnancy in a less exclusive way by disembodying them from the fetus through a fetal representation that erases them and their body. Similarly, three other papers (Taylor 2002; Stormer 2003; Verbeek 2008) highlighted how sonograms depict the fetus independently from the pregnant person, visually separating the pregnant person and the fetus, as they erase the former’s body (placenta, vascular system). According to Edvardsson, the consequence of this depiction is that the pregnant person loses her central role in the pregnancy (Edvardsson et al. 2014; Edvardsson, Mogren et al. 2015a). In this context, pregnant people end up becoming just an environment, a site in which the fetus is living, rather than a unity with it (Petchesky 1987; Zechmeister 2001; Verbeek 2008), an environment that is potentially harmful due to unhealthy lifestyle choices like smoking or drinking (Zechmeister 2001).

#### Discrimination, Selection, and Sex Determination in Screening

Some papers, especially among those that discussed the ethics of ultrasound screening for fetal abnormalities, disabilities, or other traits (such as sex), raised some concerns about the societal consequences that the routine use could have in terms of eugenic practices, discrimination, and abortion. For instance, Edvardsson, Mogren et al. (2015a) argued that ultrasound screening is pushing society towards only expecting “perfect” babies to be born and thus reducing society’s acceptance of disability or diversity. According to the retrieved literature, this tendency towards obtaining the perfect child is leading to a subtle societal pressure to use abortion to prevent the birth of children with disabilities or malformations (Yuen et al. 2006; Phutke et al. 2019) and could also, in turn, increase the number of abortions for trivial reasons—including minor aesthetic conditions such as cleft palate or the baby being of the unpreferred sex (Gottfreosdottir and Bjornsdottir 2010).

In this context, five papers specifically discussed the issues stemming from sex identification and selection through ultrasound. According to these papers, sex selection is amplified by the use of ultrasound technologies (Phutke et al. 2019) and misuse of ultrasound for the purpose of sex determination with subsequent abortion has been an area of concern in some countries (Taylor 2002; Mubuuke 2011; Holmlund et al. 2020), with a prevalence of abortions in case of female fetuses (Kongnyuy and van den Broek 2007; Edwards and Thomson 2012). Other papers described how we are entering an ethically challenging “grey zone,” where minor abnormalities and an increasing number of traits can be identified, thus setting the stage for selection, “engineered babies” (Edvardsson et al. 2018), and eugenic ideologies and practices (Hemminki et al. 2000; Favre et al. 2009; Åhman et al. 2019).

#### Personification of the Fetus and Fetal Ontology

Other relevant ethical issues highlighted by the literature were related to the construction of fetal ontology and the capacity of ultrasound to personify the fetus into an individual patient / human being. According to some papers, the fetus, through ultrasound visualization, has come to be treated as a patient with individual health interests and in need of individual medical attention (Petchesky 1987; Zechmeister 2001; Stormer 2003; Edvardsson, Mogren et al. 2015a; Favaretto et al. 2020). Even more, according to some other papers, obstetric ultrasound allows for new and developing social constructions of fetal life and fetal relationships (Chervenak and McCullough 1996; Mills 2005). As such, it is assisting in the conceptualization of the fetus as an ontologically separate person in its own rights (Sandelowski 1994; Verbeek 2008) as a baby/person/human (Taylor 2000; Sullivan 2002) or as an individual life (Taylor 2002) from a very early stage of pregnancy, thanks to the advancements in ultrasound that can act as a window into the pregnant person’s womb (Gorincour et al. 2011; Edvardsson, Mogren et al. 2015a).

In this context, the retrieved literature argued that the visualization of the fetus through ultrasound might result in the perception of the human fetus as more real (Giebel 2020). Through its technologically mediated appearance, the fetus is presented as a being towards which we establish an ethical and social relation that demands ethical responsiveness—empathy, protection, and so on. (Mills 2005; Mills 2008; Mills 2018). In addition, according to Boucher (2004), these technologies are creating a revolution not only in pregnant people’s experience of pregnancy but also in how society imagines the fetus, by conflating the image of the ultrasound with the “reality”/“personhood” of the fetus. According to a few authors (Zechmeister 2001; Mills 2008; Mills 2018; Favaretto et al. 2020), the sophistication of 3D imaging technologies and increased quality in the visualization of the fetus could have the effect of making the fetus more real and more human to the observer, by allowing the viewer to clearly see and interpret fetal behaviour as human/person behaviour.

#### Pre-Abortion Ultrasound

A few of the retrieved papers focused explicitly on the ethical issues embedded in the practice of pre-abortion ultrasound, a mandatory procedure in some parts of the United States where a pregnant person seeking an abortion is required to receive information on accessing ultrasound services or undergo an ultrasound before an abortion. Two papers highlighted how mandatory pre-abortion ultrasound forces pain and emotional distress on pregnant people by showing the image of the fetus before abortion (Graham et al. 2010) without medical indication of a necessity of ultrasound (Charo 2014). According to the literature, mandatory pre-abortion regulations clearly violate the principle of respect for autonomy (Rocha 2012). This is because they introduce coercion into the informed consent process (Steinauer and Sufrin 2014) by not giving pregnant people a choice of what information they want to have from an examination (Minkoff et al. 2004).

According to Sanger (2008), the statutes that require such a procedure are meant to transform the fetus from a simple abstraction to a real baby in the eyes of the aborting pregnant person, which could make decision-making increasingly difficult for them, since looking at the image and being emotionally affected by it could affect their decision (Graham et al. 2010). Finally, one paper highlighted one issue of this practice from the point of view of providers that might not be comfortable with performing the scan and seeing the fetus before an abortion (Graham et al. 2010).

#### Ethical Issues for Low-Income Countries

Some of the papers focused on the specific issues of the development and diffusion of ultrasound in low-income countries. A number of those described issues of monetary exploitation of patients for ultrasound screening appointments. In this context, private clinics would provide repeated and clinically useless ultrasound examinations in order to increase their profits (Kongnyuy and van den Broek 2007; Holmlund et al. 2020) by exploiting pregnant people’s lack of appropriate information about the reasons for and recommended frequency of ultrasound (Bashour et al. 2005). One paper also highlighted how monetary gain was also at the core of performing specific screenings, such as sex identification (Mubuuke 2011).

Concerns regarding overestimation of diagnostic capabilities of ultrasound were also described. According to the literature, both pregnant people and providers would have a tendency to put unwarranted faith in ultrasound at the expense of other crucial non-technological procedures such as history taking and physical examination (Kongnyuy and van den Broek 2007) and antenatal care surveillance that could easily detect serious conditions (Holmlund et al. 2020). Finally, two papers discussed issues related to access to ultrasound technology. One paper focused on the exposure to non-standardized care of part of the population, due to privatization of healthcare in Vietnam (Gammeltoft and Nguyen 2007), while the other highlighted the issues deriving from having part of the population exposed to unequal care due to lack of access to ultrasound technologies in rural parts of India (Phutke et al. 2019).

#### Keepsake Ultrasound

Nine papers focused on the ethical issues raised by keepsake ultrasound—that is, those ultrasound sessions performed for the entertainment of the parents that do not embed any diagnostic evaluation. The main concern raised by the literature in this context were safety issues related to the exposure of the fetus to ultrasound waves, especially since entertainment ultrasound sessions usually entail increased exposure time (Burlbaw 2004; Voelker 2005; Coles 2007; Simonsen et al. 2008; Raucher 2009; Yin and Che 2009). A couple of papers stressed this concern by highlighting that it is difficult to assess the biological risks of ultrasound waves and there is still uncertainty about the long term effects of such an exposure (Paul and Nawrocki 1997; Dowdy 2016).

According to the retrieved papers, there are also issues related to information and pregnant people’s knowledge about prenatal ultrasound that are exacerbated by the keepsake ultrasound setting. For instance, two papers argued that pregnant people are often not informed about the risks, limitations, and capability of ultrasound, and therefore it becomes difficult for them to make an appropriately informed choice about entertainment ultrasound (Yin and Che 2009; Dowdy 2016). In addition, providers working in non-clinical settings are not trained to inform pregnant people when something is wrong in the ultrasound, like the discovery of a fetal malformation (Voelker 2005; Raucher 2009), and they cannot initiate appropriate counselling procedures (Chervenak and McCullough 2005).

This could in turn provoke a—sometimes unwarranted—sense of concern and fear in pregnant people, when the pictures looks odd or possible malformations are detected (Chervenak and McCullough 2005; Raucher 2009), or it could even leave pregnant people with a false sense of security if they misinterpret keepsake imaging sessions as a medical examination (Paul and Nawrocki 1997; Raucher 2009). In addition, Raucher (2009) argues that inaccurate findings during keepsake ultrasound might involve the risk of having pregnant people undergo unnecessary and sometimes risky follow-up procedures. Three papers raised the issue that offering keepsake ultrasound services in the clinical setting for a fee could raise economic conflict of interests between the physicians and their patients (Chervenak and McCullough 2005; Voelker 2005; Dowdy 2016).

In addition, Chervenak and McCullough (2005) caution about the introduction of keepsake imaging services into the clinical setting, as it could increase the burden for pregnant people that were referred to ultrasound because of adverse findings or in case of at risk pregnancies. Coles (2007) argues that entertainment ultrasound creates ethical issues as it implies the invasion of the fetus’ privacy with a voyeuristic and consumeristic eye. Finally, according to some of the authors (Burlbaw 2004; Voelker 2005), 3D and 4D imaging, since they are capable of delivering more realistic ultrasound pictures to parents, are increasing the number of requests for entertainment imaging, thus exacerbating the issues described above. In addition, according to Dowdy (2016), 3D imaging might convey fetal abnormality to an untrained eye, either real or not, and cause shock or anxiety in expectant parents within a non-clinical environment.

## Discussion

This review provides a comprehensive mapping of a multifaceted plethora of ethical issues related to prenatal ultrasound. Many of the outlined issues conflict with common principles of biomedical ethics (Beauchamp and Childress 2001), such as reducing the risk of harm, ensuring informed consent, and respecting autonomy. At the same time, many of the retrieved papers went beyond such principles and discussed how ultrasound is increasingly taking a central role in pregnancy management (Åhman et al. 2019), impacting pregnant people’s role within and experience of pregnancy, and assisting in shaping fetal ontology and the moral status of the fetus.

The most prominent ethical issues highlighted by the literature are those related to counselling and information provision. In this context, our results suggest that, since its introduction in the obstetric practice, ultrasound has seldom been accompanied by appropriate counselling practices and appropriate information about the risks, benefits, and outcomes of the examination (Lowe et al. 1998; Favre et al. 2009; Thomas et al. 2017). This lack of information provision could be explained by the non-invasive nature of the procedure and the context in which the examination is taking place (Abramowicz 2013). It is in fact not uncommon for expectant parents to perceive ultrasound, not only as a diagnostic examination but also as moment to see and bond with the fetus (Kohut et al. 2002). Previous studies have in fact identified obstetric ultrasound as a “hybrid practice” where providers and expectant parents construct the social meaning of the fetus (Taylor 1998). In this context, numerous studies, especially at the beginning of the diffusion of ultrasound practices, have focused on understanding how and to what extent ultrasound examination could enhance bonding between the pregnant person and the fetus (Lumley 1990; Black 1992; Roberts 2012). Our results, however, have also highlighted that there are important negative aspects and possible harm that could derive from ultrasound, from increased anxiety for family members to the development of difficult decision-making scenarios in case of adverse findings for which expectant parents are not adequately prepared (Whitbeck 1987; Van der Zalm and Byrne 2006).

Of course, strictly connected to counselling and information are issues of autonomy. Pregnant people, if they are not appropriately informed and if they do not receive appropriate counselling, will not be able to actually make autonomous choices regarding ultrasound examination and further prenatal tests. According to Nakou (2021), the routine use of prenatal screening historically expanded as a response to pregnant people’s demands to enhance their autonomy, by improving their reproductive choices, promoting their informed choices, and managing their pregnancies. However, as highlighted by our results, societal and technological developments of this technology ended up depriving patients of their autonomy, either by creating societal pressure towards undergoing ultrasound examination (Edvardsson et al. 2018) or circumscribing their choices to a limited number of options (Favre et al. 2009; Åhman et al. 2015).

As our results show, decision-making might also be hindered by the absence of, or inappropriately provided, information. This is because ultrasound has the potential to generate huge amount of information about the conditions of the fetus in utero (Edvardsson et al. 2016; Edvardsson et al. 2018; Phutke et al. 2019) that might create difficult decision-making scenarios for parents, from proceeding with invasive testing (Verbeek 2008) to elective termination of pregnancy (Taylor 2002). All of these issues only highlight the importance that patients are adequately informed about the aims, complexities, and possible outcomes of ultrasound (Ahman et al. 2010). At the same time, however, some studies have reported the difficulties and challenges that providers encounter facing the task of appropriate counselling. It has been argued in fact that it is difficult to even decide what would constitute appropriate information for patients (Edvardsson et al. 2014; Edvardsson et al. 2016) and that, due to the limited time providers are allowed to grant to each ultrasound session, appropriate information provision before the examination might take too much time away from the actual exam (Åhman et al. 2019). Counselling has been described as a challenging “balancing act” (Edvardsson et al. 2018), where providers need to consider (weigh, balance) the information they extract from the ultrasound and parents’ expectations and worries.

This contradiction, highlighted by our review, only emphasizes the importance of developing strategies for informing obstetric patients in a way that is compatible with the time and expertise of providers and the expectations and knowledge of patients (Howe 2014; Åhman et al. 2015). The *WHO Recommendations on Antenatal Care for a Positive Pregnancy Experience* states that “before performing the ultrasound examination during antenatal screening, the provider should counsel the woman on potential benefits and limitations of the scan” (World Health Organization 2018). However guidelines or indications on how to appropriately provide such information is not furnished by the document. Appropriate models for counselling could include the development of training for providers to promote consistency in counselling in different scenarios (Edvardsson et al. 2016) and to offer them strategies to avoid information overload but still allow well-informed decision-making (de Jong et al. 2011). Another interesting suggestion could be to dedicate an appropriate time before the examination, in a language and detail that is compatible with the patients’ understanding, where providers explain the purpose of prenatal ultrasound, the information that might be discovered through the examination, and the degree of certainty of such information (Lowe et al. 1998; McFadyen et al. 1998; Thomas et al. 2017). By enhancing counselling and taking the time to inform patients, patient autonomy and informed choices could be adequately fostered.

The results of our review also show how autonomous choices and decision-making in prenatal care might also be strongly influenced by ultrasound examination. This might be induced by the nature of the examination in itself, which embeds the capacity to show the fetus (Zechmeister 2001; Favaretto et al. 2020). Fetal revelation and visualization might have an impact on decision-making on two levels. The first is by visually separating the pregnant person and the fetus (Brauer 2016), thus promoting the conceptualization of the fetus as an ontologically separate entity or an autonomous subject, with a singular meaning. As Mills describes, ultrasound images “put us in relation to a being that we do not otherwise have such a relationship with” (Mills 2011) and, thanks to the advancements in technology, these images are becoming more realistic, thus granting more realism to this being as an individual and as a patient. This personification of the fetus through the creation of fetal images becomes intrinsically problematic when considering the interests of the pregnant person and the fetus both when such interests are aligned, as one might overlook that the two sets of interests are inextricably linked (Duden 1993; Kukla and Wayne 2018) and when they are at odds, as pregnant people’s rights might be obscured or overruled (Samerski 2015; Frost and Haas 2017).

The other is by (visually) exposing the fetus as a moral agent (Brauer 2016; Mills 2018). Again Mills argues that not only ultrasound makes possible the social appearance of the corporeal life of the fetus, it also establishes a demand for ethical response (Mills 2005). Within this ethical dimension, the pregnant person and the provider would have a responsibility to answer to the demands of the fetus, creating here as well issues in case of conflicting health interests between the pregnant person and the fetus. Such ethical response could also explain the reasoning behind practices like mandatory pre-abortion ultrasound screening (Charo 2014) or the extensive use of fetal imagery in antiabortion campaigns (Gilbert and Howes-Mischel 2004).

Images are powerful objects, loaded with social and emotional significance. As shown by our results, fetal images might influence or have a profound ethical implication on society’s perceptions of the status of the human fetus by shifting our attitudes toward the subjects they represent. As such, we need to be careful on how these images are used, shown, and presented to both patients and society. Consequently, a thorough and careful examination of the societal impact of these images needs to be initiated and updated, especially in light of technological advancements that make the image of the fetus so realistic. Finally, it would be extremely relevant to analyse the phenomenology of 3D ultrasound scans to investigate how their visualization relates to ethical responsiveness and to analyse if and consequently how the realistic observation of the fetus through the image can change our attitude towards it.

Another interesting result is the entwinement between the social and diagnostic value and the spectacularization of ultrasound practices, in the form of entertainment ultrasound. As our results show, there are numerous issues related to exclusively keepsake ultrasound practices, such as informed consent (Raucher 2009; Dowdy 2016) and possible harm to either the fetus or the pregnant person (Chervenak and McCullough 2005; Coles 2007). In addition, our results also highlighted the concern of certain authors that current ultrasound practices are spectacularizing pregnancy and obstetric care (Sandelowski 1994; Sandelowski 1998) and that ultrasound technologies are becoming more and more a technology for social and parental entertainment rather than a diagnostic tool (Sullivan 2002). This dichotomy between the diagnostic versus entertainment use of ultrasound might also explain why concerns of fetal harm and physical risks associated with ultrasound were much more prominent in the literature on keepsake ultrasound, rather than in the literature where the diagnostic use of ultrasound was considered, where such concerns were more related to the difficulty of balancing ultrasound’s risks and benefits. It is undeniable that keepsake ultrasound practices and services are to some extent aggravating the perception of ultrasound as entertainment and spectacle; at the same time, however, it has been argued that the social aspect of ultrasound should not so easily be discarded as mere entertainment.

Some scholars have in fact underlined how the value of ultrasound practices exceed clinical utility for families and pregnant people (Gudex et al. 2006), describing them as an important moment that allows for the social construction of fetal relationships (Chervenak and McCullough 1996; Mills 2005) and a resource to begin to construct the social identity of the fetus—as an individual that bears resemblance to the other members of the family (Taylor 2008). Kroløkke (2011), for instance, interestingly argues that the ultrasound examination room can be interpreted as an arena in which expectant parents and family members rehearse their new identities. As such, although less explored than clinical dimensions, it seems that wider familial implications of ultrasound might be significant for expectant parents (Roberts et al. 2015). We should therefore not discard the discourse around entertainment examination as a mere consumeristic practice, as it is embedded within a more complex social dimension. We should therefore start interrogating ourselves whether current obstetric practice should reconcile the social and diagnostic role of ultrasound (Simonsen et al. 2008) and if this is a task that should be undertaken by medicine and within the hospital/clinical setting.

Lastly, our results show how the development of 3D and 4D ultrasound are currently exacerbating the issues related to entertainment ultrasound and the increased spectacularization of obstetric practice (Sullivan 2002). However, very little research has actually focused on analysing the new social dimensions that this technological development is raising in the obstetric practice (Roberts 2012) and its impact on the dissemination of ultrasound keepsake practices. Also, in general, as our results point out, there is very little literature that discusses the ethical issues of 3D and 4D ultrasound. It would be therefore extremely relevant that future studies analyse the value that obstetric practitioners and expectant parents bestow on 3D and 4D ultrasound in order to understand the role that this technology is adopting in obstetric practice, including the specific ethical and societal issues it might raise.

## Limitations

In this systematic review, a total of seventy-two peer-reviewed papers in English that qualified for inclusion were assessed. The authors have also left out the grey literature to focus exclusively on peer-reviewed academic papers. It might thus be possible that studies in other languages and relevant grey literature have been overlooked.

## Conclusions

Our systematic review highlights the most pressing ethical and societal dimensions that the sophistication and routine use of prenatal ultrasound is raising for the obstetric practice, pregnant people, and fetal ontology. Our analysis highlights how issues of consent, information provision, and counselling are not only the most prominent ethical concerns related to prenatal ultrasound, but also critically impact pregnant people’s autonomy and their role within pregnancy, bodily experiences, and choices. Consequently, we argue that implemented counselling and information provision strategies should be developed to appropriately inform pregnant people about the nature, purpose, and choices revolving around ultrasound. Moreover, our review highlighted how pregnant people’s autonomy and decision-making are strongly influenced by the personification of the fetus, both as a patient and a person, that is being exacerbated by the visualization and depiction of the fetus provided by the sophistication of ultrasound technologies, including the development of 3D ultrasound. Finally, our analysis underlined how, thanks to the development of ultrasound imaging and the practices of keepsake ultrasound, it is becoming increasingly difficult to separate the social and clinical dimensions of prenatal ultrasound. As a consequence, future research should focus on, first, achieving clarity regarding the risks and benefits—both social and medical—related to prenatal ultrasound and, second, examining whether the two dimensions could and should indeed be reconciled.

## Data Availability

Underlying data is publicly available—that is, included studies can be obtained by readers. Extracted data can be made available by the corresponding author upon reasonable request.
